# Tool making cockatoos adjust the lengths but not the widths of their tools to function

**DOI:** 10.1371/journal.pone.0205429

**Published:** 2018-11-07

**Authors:** A. M. I. Auersperg, C. Köck, M. O’Hara, L. Huber

**Affiliations:** 1 Messerli Research Institute, University of Veterinary Medicine Vienna, Medical University of Vienna, University of Vienna, Vienna, Austria; 2 Department of Cognitive Biology University of Vienna, Vienna, Austria; Consiglio Nazionale delle Ricerche, ITALY

## Abstract

The ability to innovatively use or even manufacture different tools depending on a current situation can be silhouetted against examples of stereotyped, inborn tool use/manufacture and is thus often associated to advanced cognitive processing. In this study we confronted non-specialized, yet innovative tool making birds, Goffin’s cockatoos (*Cacatua goffiniana*), with an apparatus featuring an out-of-reach food reward that could be placed at different distances from a tool opening. Alternatively, the food stayed at a constant distance but the tool opening in the front of the apparatus had different diameters. We used a novel material for tool manufacture (cardboard) that demanded an incrementally increased manufacturing effort from the actor, depending on the length of the tool required. We found that our subjects used two strategies to succeed in this tasks: either by making carboard-stripe tools using the full length of the material sheets originally offered or by adjusting the lengths of their tools to different goal distances. Subjects also discarded cardboard stripes that were too short to reach the goal prior to use and discarded longer pieces when the goal was further away than when it was close. Nevertheless, likely due to morphological constraints, the birds failed to adjust the widths of their tools depending on the diameter of the tool opening.

## Introduction

While tool use and manufacture may result from inflexible and/or hereditary determined behavioural patterns, tool behaviour that can be swiftly adjusted to a changing environment requires cognitive investment and is usually linked to innovation and creativity [1, 2]. Previous studies emphasized a correlation between enlarged brain sizes and technical innovations including tool use (see e.g. [3–7]). Furthermore, the plastic performance of some tool-using animals in technical tasks suggests an elementary understanding of some of the physical characteristics underlying the respective problems ([1, 8] for summaries).

Outside primates (e.g. [9] and more), the ability to adjust tool use and manufacture to task requirements has been most systematically studied in the New Caledonian crow, which is probably one of the most proficient animal tool users [10]. For example, Chappell and Kacelnik [11] tested two crows in a task featuring choices between sticks of different lengths to retrieve an out-of-reach food reward positioned at different distances from the opening of a horizontal tube. Indeed, it was found that the birds selected tools matching the distance to the food significantly more often than would be expected by chance. Furthermore, simply choosing the longest tool (which was always functional) also appeared to represent an important strategy [11]. When another habitually tool using species, the woodpecker finch was confronted with a similar tool-length-decision task, the majority of subjects were also able to reach the food reward with the first tools they picked. Nevertheless, other than the crows in the previous study, they did not match the tools to the given distance [12]. Like the crows, the finches tended to choose tools that were longer than necessary but had no pronounced preference for the longest ones. Interestingly, when adult humans were confronted with setups as used by Chappell & Kacelnik [11], they neither matched the exact distance nor did they always select the longest stick. While the length of the stick correlated with the position of the food, humans tended to choose relative to the lengths of the alternative tools [13].

As the previous experiments indicate, selection tasks testing the animal’s apprehension of the relationship between tool length and food distance can be limiting as choices between two or more tools could also be quickly accomplished by applying simple rules. This can make it harder to interpret the animals’ performance. For example, subjects can circumvent the problem by continuously selecting the longest tool (which is always functional) without suffering any significant costs.

Tool manufacture is rare, even amongst tool using animals [8]. Nevertheless, once an available option, structural adjustments prior to tool use can be useful to investigate plasticity in tool related behavior as they can help us to reveal the animal’s perception of a tool’s functional properties. Furthermore, failing to make a functional tool is costlier in terms of time and effort than merely making an uninformed choice. If we take the previous example, simply manufacturing a longer tool can sometimes come at higher costs as the stem of a longer twig may be naturally thicker at its base and may thus be harder to break [14] or the making of the longer tool may involve more steps than making a short tool [15]. Differences in the number and/or type of steps between the manufacturing processes and the appearances of the tools required for different tasks may help to increase the amount of meaningful information we can deduct from the animals’ performances.

Alongside primates, tests on tool adjustments to different conditions have largely been focused on New Caledonian crows (aside from recent research on the focal species of this study). When crows were presented with three sticks, one loose and the other two in a bundle, they only undid the bundle if it contained the required tool. Furthermore, the crows detached different tools from twigs, depending on the diameter of the opening through which the tool needed to be inserted [16]: they mostly used tools that were narrow enough to fit into the opening. Moreover, the maximum diameter of the ‘working’ end of their tools increased with the diameter of the tool opening. It was also shown that the same birds could make two completely different tools by bending or unbending pliant material depending on task demand [17]. Hunt et al. [18] investigated how two New Caledonian crows manufactured tools to extract food from vertical holes of different depths. They found no evidence that the crows made the lengths of their first tools to directly match hole depth. However, while the first applied tools were of a similar length regardless of the hole’s depth, successive tools were significantly longer if the food distance was far. Hunt et al. [18] argue that the New Caledonian crows may use a two-stage heuristic strategy to solve tool making tasks: the first tool may represent a ‘default behaviour’ while the second tool is either the product of an associative learning rule based on the outcome of the default behaviour or a delayed inference to the causal properties of the task. In 2016, wild-caught New Caledonian crows were presented with a similar task [14]. Nevertheless, as the effort between making a short and a long tool was still small, in order to prevent them from simply always building long tools, irrespective of the distance of the food reward, a physical barrier was placed opposite the tool opening when the food was located at a short distance. This was done to increase the difficulty of using long tools to access the food. They found that the crows indeed made shorter tools when the food was close and longer tools when the food was distant. However, they often failed to match the tool length sufficiently to extract the food. The authors conclude that the animals most likely followed a relative rule, such as making tools longer or shorter, or always make a shorter tool when the barrier is present rather than an absolute rule, such as matching exact lengths required. As learning seemed to be involved in the task acquisition, the birds may also have experienced negative reinforcement as the apparatus intended to require only short tools was often not effective.

The Goffin’s cockatoo is a generalist, opportunist parrot (Mioduszewska, O’Hara unpublished data). It is neither a specialized tool user nor a habitual tool user on a population wide level such as the New Caledonian crow [10]. They are also neither nest builders nor food cachers, which are believed to be important preconditions for the development of avian tool use [19–21]. Nevertheless, they combine and modify environmental objects during exploration and play [22], which can cause individual tool innovations in captive and potentially also in feral individuals [23, 24]. At least captive birds have been shown capable of socially transmitting tool use across individuals and tool-using birds can thereafter innovatively acquire tool manufacture [25]. Additionally, these birds show great flexibility in decision making tasks featuring binary choices between two completely different tools: They are capable of adjusting their choices relative to the quality of the reward, the amount of effort involved and in the functionality of the respective tool with the apparatus at hand [26, 27].

We recently learned that Goffin’s cockatoos can innovatively manufacture the same (long stick-type) tool from different material sources [15, 23, 28]. Two subjects were able to fabric such tools from materials that required active shape-giving, such as cardboard [15]. While doing so they placed a large number of parallel bite marks into the edge of a sheet of cardboard and curved the tool off the original sheet after it reached a certain length. While cardboard tool manufacture is expected to be energetically costly (likely costlier than simply breaking off a twig) due to the number of bite-marks that need to be actively placed, the curving-off technique allows for a relatively exact length adjustment. Notably, in the prior experiment, the cardboard tools were only about one centimeter on average above the minimum length (6 cm) required to reach the food reward. In contrast, tools made from larch wood that broke along the age lines of the tree and therefore did not require active shaping were significantly longer, usually the full length of the material provided [15].

While the previous data suggests that subjects may attend to tool length when making such tools, we were unable to corroborate this as the distance of the food reward to the tool opening was unchanged across conditions [15].

With this study we thus aim to address two main research questions: Can Goffins adjust the properties of their tools to save effort? If so, how accurately do they attend to the properties of their tools relative to their respective function during tool manufacture? By measuring not only successful/unsuccessful tools that are inserted into the box but also pieces of material that are discarded before insertion we intended to gain important insights into different mechanisms underlying their adjustment behavior.

The cardboard material offers a valuable means to investigate stick tool adjustment: due to the number of parallel bitemarks that need to be placed, the energy that is necessary to make a stick-type cardboard tool gradually increases with the length of the material which allows for a relatively clear measure of effort. Furthermore, it theoretically allows the animals to not only manipulate the length but also the breadth of a stick-type tool. As previous studies on tool use/making adjustment in habitually tool using birds [14, 16, 18], we thus plan to manipulate the distance of the goal and the diameter of the tool-opening, but we use cardboard blocks as the material offered for manufacture.

While all our focal subjects have previously exhibited the capacity of making appropriate decisions in tool selection tasks [26, 27], at least one bird seemed to be able to switch between making hooked or straight tools depending on task demands by bending pliant materials [28]. Given these observations and suggestive evidence from a previous study [15], we predicted that the animals are capable of saving effort by adjusting the length of manufactured cardboard tool, at least to some extent. We also presumed that tools that are applied to the apparatus are predominately above the minimum length to reach the food for each condition. Further, we expected that inadequate (too short, too wide) tools should be discarded before being applied to the task. We take into account that several (not mutually exclusive) strategies may underlie successful outcomes. Aside from higher cognitive explanations, associative learning or possibly a two-stage heuristic strategy as found in New Caledonian crows [18] could be necessary. Another scheme might be a general preference for longer tools as in New Caledonian crows [11] or woodpecker finches [12], if the tradeoff for larger tool construction is not high enough.

Finally, we predicted that subjects would not make wider stripes if the opening diameter is wider than usual and only make thinner stripes if the usual tool width does not fit the opening. Making slimmer tools may also require them to switch to another tool making technique (if they still used the same technique for making regular cardboard tools as in previous studies, the breadth of their tools might be partly constrained by their beak morphology [15]).

## Materials and methods

### Subjects

Six adult Goffin’s cockatoos (5 males and 1 female) participated in this study. They were hand-raised and housed in a large, enriched group aviary (indoor: 45 m^2^ ground space, 3–6 m high wall to gable; outdoor: ca. 200 m^2^ ground space; 3–4.5 m high) in Lower Austria (an outpost of the Messerli Research Institute of the University of Veterinary Medicine, Vienna). The indoor aviary was kept at 20 C° during the winter and was set at 12:12 h light dark cycles. Fresh drinking water and various fresh and dried fruit, vegetables, seeds, protein and mineral sources were available ad libitum (certain treats such as nuts were only used as food rewards during experimental routines). Subjects were marked with colored aluminum bands for individual identification. All birds were adapted to a daily experimental routine and observation. They were tested individually in an adjacent indoor testing compartment (7.5 m^2^, 3 m high) in visual isolation from the rest of the group.

All subjects had previous experience in diverse tool using tasks, including the use of stick-type tools [15, 23, 25–28]. As mentioned in the introduction, two subjects (the adult males Dolittle and Figaro) had manufactured cardboard tools before but neither the position of the reward nor the diameter of the tool opening had been altered in the previous study [15].

### Ethics

The animal keeping conditions comply with the species-specific guidelines for parrots provided by the Austrian Animal Protection act. Furthermore, the keeping facility and the animals are registered at the local Animal Welfare Bureau (Bezirkshauptmanschaft St Pölten, NÖ). All subjects were acquired from European breeders in compliance with CITES regulations.

Since the presented experiments are purely appetitive and strictly non-invasive they do not qualify as ‘Animal Experiments’ according to Austrian Law. Nonetheless, this study was discussed with and approved by the institutional (University of Veterinary Medicine, Vienna) ethics and animal welfare committee in accordance with good scientific practice guidelines and national legislation. We would like to add that since none of our birds were clipped, participation in the experiment was voluntary. Either the experimenter entered the group aviary and asked the subject to step up on the hand in order to bring it into the experimental compartment, or the door to the testing compartment was opened and the respective bird was called in by name. We did not have motivational problems in this study.

### Apparatus

**Fig 1:**
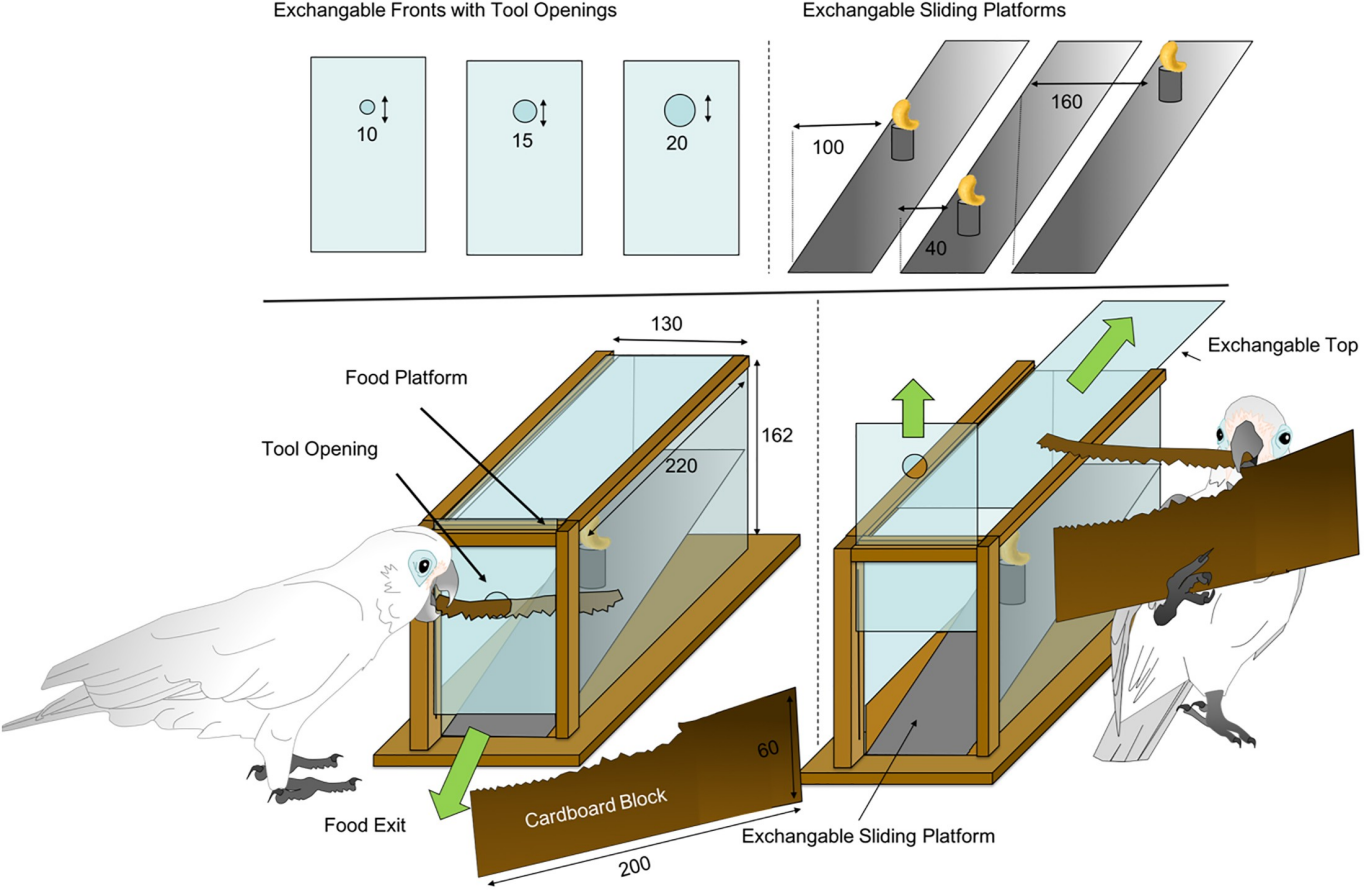
Basic apparatus with exchangeable fronts and platforms (dimensions in mm).

The apparatus was a long rectangular and transparent box (see Fig 1 for dimensions) bearing a reward platform fixated on a sliding (slanted) plate. The sliding plate, as well as the Plexiglas front were exchangeable. The reward platforms on the sliding plate could be positioned at three different distances (4, 10 and 16 cm) from the frontal hole in the box and the frontal hole came in three different diameters (1; 1,5 and 2 cm ∅). The apparatus was attached to a heavy wooden board (35 x 35 cm) to prevent the birds from tilting it over. A cardboard sheet (training: “Bristolkarton” 15 x 6 cm; thickness: 1,0 mm; testing: “Finnpappe”, 20 x 6 cm, thickness: 1,0 mm) was placed in front of the apparatus.

### Training

All of our subjects were proficient stick tool users at the beginning of this experiment. Two birds (the adult males, Dolittle and Figaro) had previously manufactured tools from cardboard [15]. Four additional birds (female: Fini, males: Kiwi, Konrad, Pipin) were trained to do so (see S1 File).

### Testing procedure

Prior to testing, each subject received one habituation session (consisting of 10 trials) to the new, unfamiliar apparatus, alongside a cardboard block (15 x 6 cm) for tool manufacture. For the habituation, the food platform and the frontal tool opening were set at our baseline arrangement (baseline distance of food platform = 10cm, baseline frontal opening size = 1,5 cm ∅).

For all testing sessions, the subjects were offered slightly longer cardboard blocks made out of a tighter material (20 x 6 cm).

We used two subtasks in this experiment: a distance task (DT) and a width task (WT). In the DT the diameter of the Plexiglas hole stayed at baseline (1,5 cm ∅), while the distance of the food platform varied (two conditions: close reward = 4cm; far reward = 16 cm). Whereas in the WT the distance of the food platform remained at baseline (10 cm) while the diameter of the frontal hole varied (two conditions: small opening = 1 or large opening = 2 cm ∅).

Each bird had to complete both tasks. Subjects received six sessions of each test (DT, WT). Within a session each condition (near/far or small/large) was offered five times in random order. Subjects were divided into two groups of three birds. Group A received DT first followed by WT and group B vice versa.

Similar as in the training, the birds were given up to 10 testing trials per session and up to 15 minutes per trial. If a bird did touch neither the apparatus, nor the cardboard for a time span of five minutes, the trial was stopped. If a trial was not successful, the remaining trials were randomized again and the subject received one motivation trial (a ready-made wooden stick; the 1,5 cm ∅ Plexiglas hole, as well as the 10 cm food platform was used) in between the testing trials. If a bird failed to retrieve the food during the motivation trial it received a new session on the next testing day. Successful cardboard tool manufacture and use was rewarded with a sixth of a cashew nut.

### Analysis

All trials were videotaped (Panasonic Camcorder Model No.: HC-V160) and coded directly from the videos. Due to methodological issues, exact measurements of discarded and unsuccessful material pieces are lacking in 5.5% of the trials (Information on successful tools is complete for all trials; see S1 File). All other cardboard pieces manufactured while testing—the successful tools, the pieces of material that were unsuccessfully inserted into the box as well as the first pieces of material that were manufactured but discarded before establishing contact with the box were measured, labelled and stored. We measured the length from the two farthest spots as well as the width at the distal end, the proximal end and at the center of the tool. The website ‘www.random.org’ was used to randomize the test-combinations (assignment of subjects into two groups; order of conditions within a trial).

To investigate success-rate of first employed tools we used a binomial mixed model with logit link function. Condition (near reward, far reward, large opening and small opening), session, as well as group (testing an effect of task order) were included as fixed effects and we accounted for repeated measures by considering subjects as a random effect.

In order to test to what extent individuals adjust the length of the tools produced to the distance of the food reward, we fitted a linear mixed model on the square root transformed length of the first pieces of materials that were inserted into the opening (successful and unsuccessful tools) and discarded pieces of material (immediately discarded before establishing contact between the tool and apparatus), smaller than the entire material block (200mm). Session, group, as well as an interaction term for condition and tool application (used or discarded) were included as fixed factors and subjects were considered as a random effect.

To investigate the number of tools with a maximum length (200 mm) used in the distance conditions we fitted a generalized linear mixed model, following a Poisson distribution. Distance to food, session and group were included as fixed effects and subject as random effect.

In each condition we tested if overall tools employed on average exceeded the required length to reach the reward using Wilcoxon 1-sample signed ranks tests with mu set to the respective reward distance.

The relation between session and tool length was tested for by employing Kendall’s Tau correlation.

To discern potential underlying cognitive strategies, we analyzed the length of first and second applied tools with a further linear mixed model including group, session and the interaction of condition and tool order as fixed factors. Both models included subjects as a random factor to account for repeated measures.

Tool width was compared only descriptively between conditions and tool application (discarded or used) for the subject Fini, who successfully solved the small opening condition. In order to explore an influence of morphological restrains (e.g. beak length determining the maximum width of created tools) on the width of created tools, we compared the width to beak measurements of tested subjects using Wilcoxon 1-sample signed ranks tests with mu set to the respective beak length.

Model selection was based on stepwise backward model refinement from a full model using the Akaike Information Criterion (AIC) and likelihood ratio testing (see [29]). Model assumptions were checked visually and where applicable normality of residuals was tested using the Shapiro-Wilk test, while the assumption of homoscedasticity was tested for using Levene’s test for each factor, or interaction of factors. Alpha levels were set to 0.05, factor level contrasts were set manually and p-values were adjusted for multiple testing employing the false discovery rate correction [30, 31]. Statistical analysis was carried out in R version 3.4.3 (R Core Team, 2017). Models were calculated using the lme4-package [32] and graphical representation of results employed ggplot2 [33].

## Results

### Analysis of performance in different conditions

**Fig 2:**
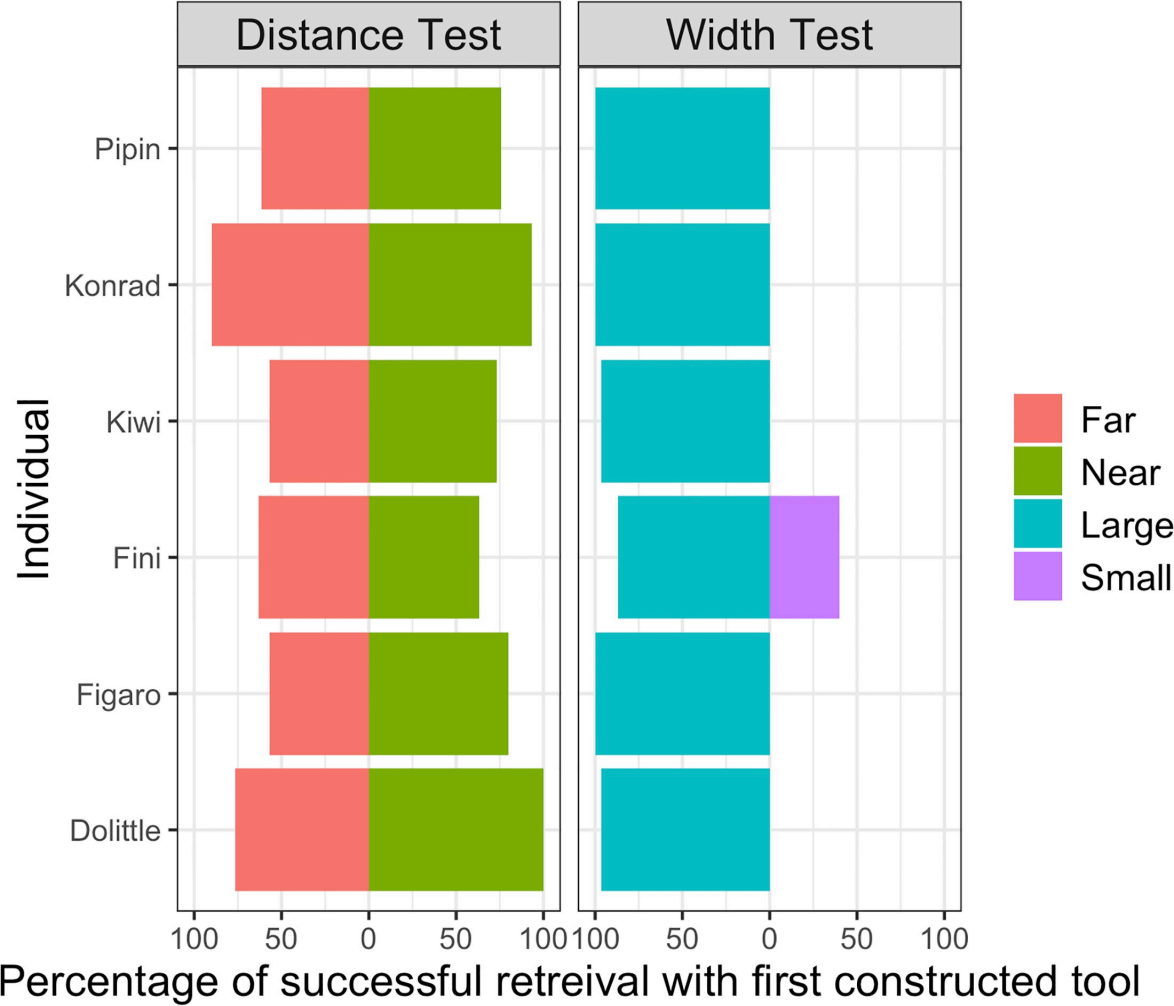
Percentage of successfully retrieving the reward with the first tool in each trial over different conditions and for each individual.

Overall, no effect of group (GLMM: *χ*^2^(1) = 0.32, p = 0.571) was found, but rate of success was affected by session (GLMM: *χ*^2^(1) = 35.43, p < 0.001; see Figure A in S1 File) and condition (GLMM: *χ*^2^(3) = 425.38, p < 0.001; see Figure B in S1 File). All four conditions differed significantly from each other (see Table A in S1 File for detailed contrast results). Within the subtest for distances the ‘far’ condition (M = 67.78% ± 4.38 SEM) was less readily solved than the ‘near’ condition (M = 81.11% ± 3.73 SD). While subjects were most successful in the ‘large opening’ condition (M = 96.67% ± 1.26 SEM), only one individual (Fini) was able to succeed in the ‘small opening’ condition (M = 6.67% ± 2.87 SEM; see Fig 2 for overview).

### Tool length

Regarding the lengths of tools applied in the distance task, our model revealed no significant effect of group (LMM: *χ*^2^(1) = 0.34, p = 0.56). Thus, the final model with best fit included session as a fixed factor and the effect of tool use nested in condition. While session influenced the model only marginally (LMM: *χ*^2^(11) = 18.85, p = 0.064), a significant effect of use nested in condition (LMM: *χ*^2^(11) = 103.57, p < 0.001) was found.

**Fig 3:**
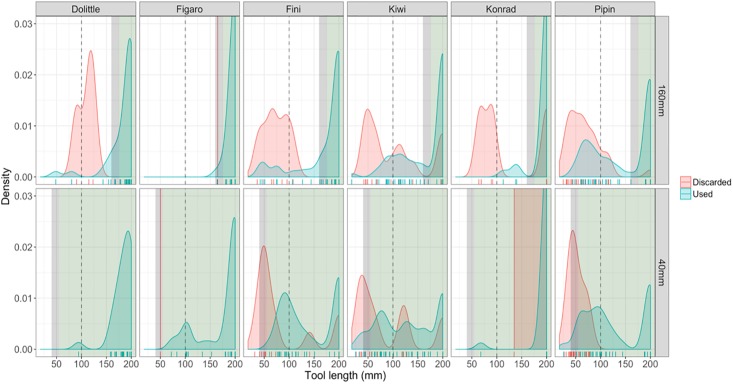
Density plots of all employed (green) and discarded (red) tools; green areas indicate potentially functional length; the gray area indicates a 15 mm margin that might be required to maintain manipulability of the tool; gray dashed line represents distance to the reward in the baseline condition and the ‘width’-test; jitter below the density plot provides information about number and exact length of tools.

Most notably, significantly longer tools were employed (first objects successfully and unsuccessfully inserted into the box) when the reward was distanced 160 mm from the opening (M = 133.5 mm ± 4.88 SEM; LMM: β = 0.72, SE = 0.26, p = 0.0269) than when it was at 40 mm (M = 118.8 mm ± 4.36 SEM) from the opening (see Fig 3). Further, discarded tools, that is, tools that were dropped before inserting them, varied significantly between the conditions (LMM: β = 1.09, SE = 0.38, p = 0.019). Shorter pieces (M = 57.8 mm ± 3.87 SEM) were discarded when the reward was closer, whereas longer pieces (M = 75.5 mm ± 4.7 SEM) were discarded in the far condition (see Figure B in S1 File). All other combinations between discarded and used tools in the different distance conditions exhibited significant differences (see Table B in S1 File for detailed contrast results and compare Figure B in S1 File).

However, considerable qualitative variation between and within subjects could be observed with peak densities of used tools towards the margins of the functional size, generally at larger (towards maximum) tool sizes and around the ‘baseline’-length of 100 mm. Discarded tools generally occur around 100mm in the 160mm distance condition, but shift towards the borders of the functional length in the ‘close’ condition for three individuals (Fini, Kiwi and Pipin) which discarded the majority of the tools. Here it is important to mention that tools of an absolute correct length (bridging the distance from hole to reward) are not necessarily of functional length (as a part of the tool must be maintained in the beak to be manipulated; the difference between absolute and functional length is indicated by grey shading in Fig 3). In the ‘far’ condition (160 mm) peak densities of tool lengths are found mainly within the functional length for three individuals (Figaro, Dolittle and Fini) and Konrad manufactured only few tools shorter than the maximum size of 200 mm that fell between baseline (100 mm) and functional length. Only one individual (Kiwi) exhibited a distribution of tool-length around the baseline distance (100 mm), while Pipin exhibited two major density peaks, producing tools either below 100 mm or within the functional length. Regarding the ‘near’ condition three individuals (Fini, Kiwi and Pipin) exhibited a shift in peak densities below 100 mm, whereas Figaro and Dolittle mainly produced tools at the baseline length or above. Konrad only produced and discarded each one used tool that was not 200 mm in this condition, which were within the functional length.

Following up the marginal effect of session on overall tool lengths revealed a significant positive correlation (τ = 0.15, p < 0.001), indicating increasing tool length in consecutive sessions (see Figure C in S1 File).

**Fig 4:**
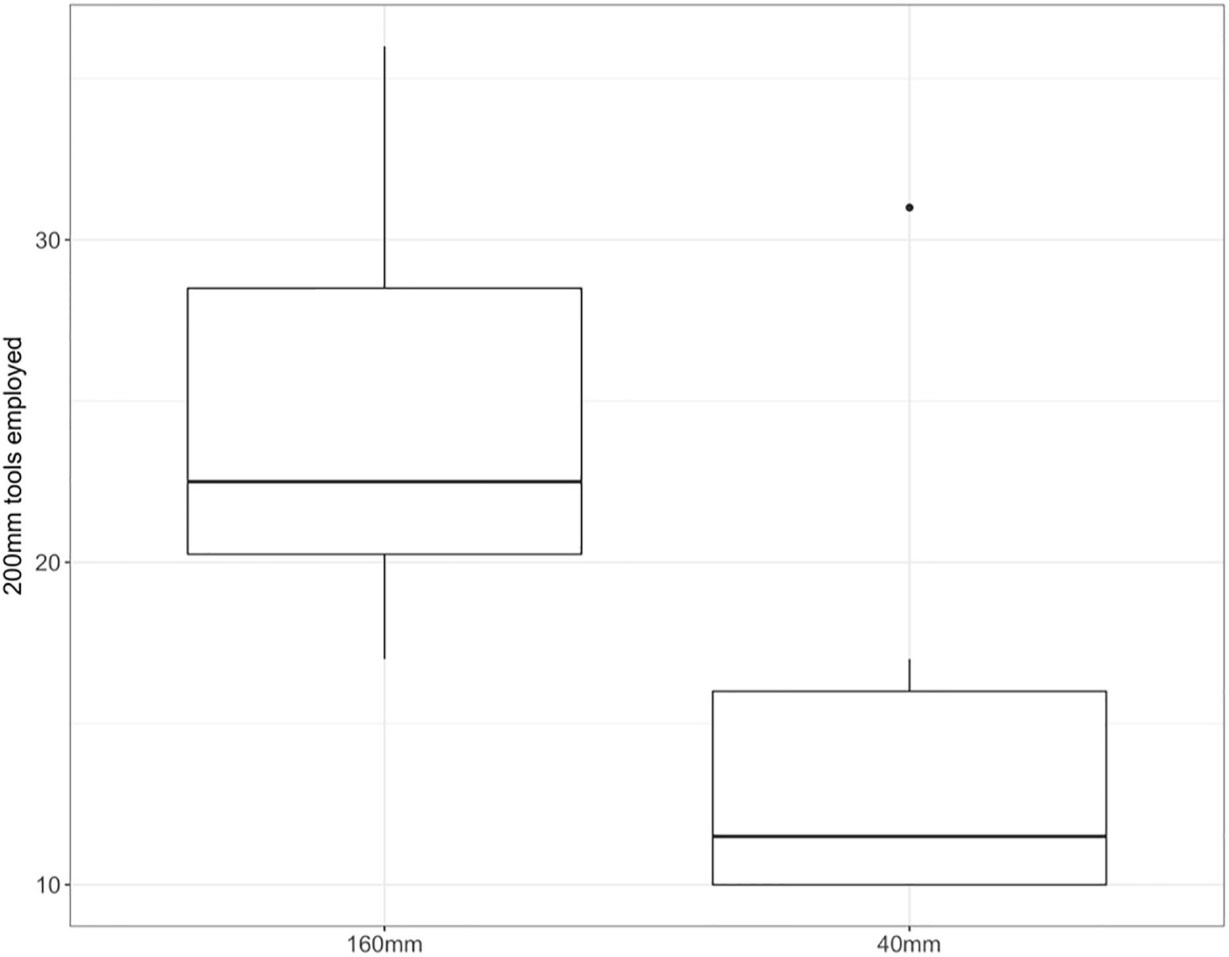
Average number of tools with maximum size (200 mm) employed in the far condition (food distance 160 mm) and the near condition (distance to food 40 mm) with small holes; bold horizontal lines indicate median values, boxes span the first to third quartiles and whiskers represent 95% confidence intervals.

Analysis of the number of tools that were not adjusted in length (using maximum length tools) in different distance conditions revealed a significant effect of distance to the reward (GLMM: *χ*^2^(1) = 8.1, p = 0.004). In the near condition, significantly fewer 200mm tools were employed (M = 2.9 ± 0.27 SEM) than in the far condition (M = 4.2 ± 0.28 SEM; GLMM: β = -0.38, SE = 0.13, p = 0.005; see Fig 4). Group and session revealed no significant effects.

On average, significantly longer tools than the distance to the reward were employed in each condition (near: M = 163.7 mm ± 3.9 SEM, p < 0.001; width: M = 177.3 mm ± 2.3 SEM, p < 0.001, far: M = 181.7 mm ± 3.1 SEM, p < 0.001).

### Relation between first and second tools

Linear mixed models regarding the length of first and second employed tools (including max size tools) in only unsuccessful first attempts, revealed a significant sequential effect (LMM: *χ*^2^(1) = 4.58, p = 0.032). Secondary applied tools were longer than tools used in first (unsuccessful) attempts (LMM: β = 20.79, SE = 9.61, p = 0.033; see Figure D in S1 File). No significant effect of distance (LMM: *χ*^2^(1) = 2.50, p = 0.114) or session (LMM: *χ*^2^(1) = 2.03, p = 0.155) was found.

### Tool width

Fini, as the only individual succeeding in the ‘small’-condition, potentially discriminated between tool width with regard to tools she discarded. Discarded tools were on average narrower in the large condition than in the small condition (large: M = 9.43 mm ± 0.55 SEM; small: M = 11.02 mm ± 0.14 SEM). While differences between inserted tools appear marginal (large: M = 9.98 mm ± 0.20 SEM; small: M = 10.52 mm ± 0.17 SEM, see Figure E in S1 File), overall tool width rather seemed to be confined by beak morphology (see below).

**Fig 5:**
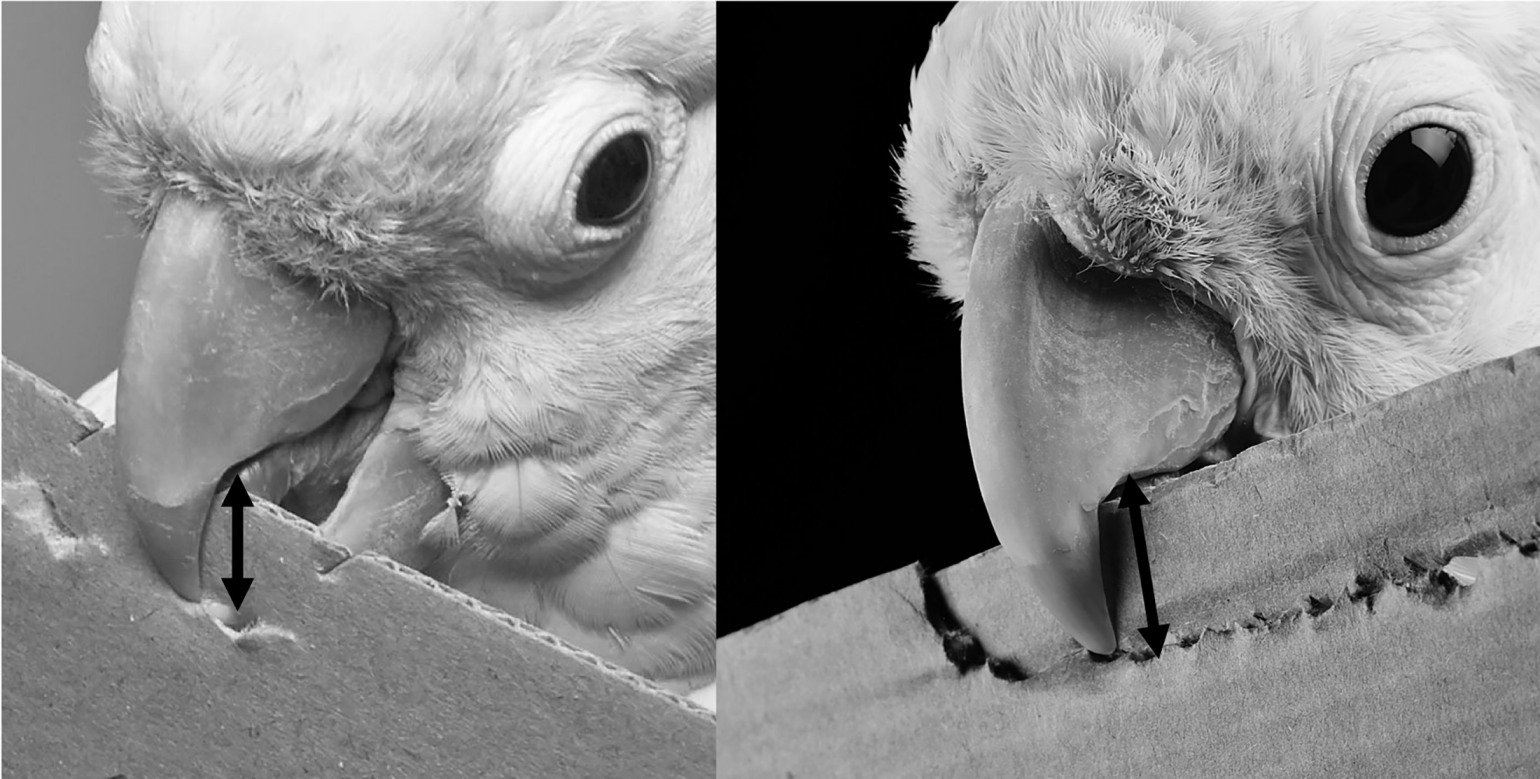
A morphological feature that is likely involved in restricting the width of a cardboard tool is the distance between the beak tip and the edge of the horizontal part of the upper mandible (left: Dolittle; photo by Bene Croy; right; Figaro; photo by Solvin Zankl): Birds used their sharp lower mandible like a cutting edge against their strong upper mandible. They placed their head ca. at a 90 ° angle towards the carboard’s side, taking the edge of the material into their beak. Cutting deeper into the material may thus cause ergonomical difficulties.

Investigating the potentially restrictive effect of beak length on the width of created tools (see Fig 5) we found that two individuals produced significantly wider tools in relation to the size of their beak: Kiwi (beak = 14 mm, Mtool = 14.3 ± 0.16, p < 0.001) and Konrad (beak = 14 mm, Mtool = 14.6 ± 0.09, p < 0.001). Note: We measured only the edge of the upper mandible from the tip to the curving point into the horizontal part as it seemed to be most likely to restrict tool width from our videos (see Fig 5). Fini exhibited no significant difference (beak = 10.5 mm, Mtool = 10.5 ± 0.1 SEM, p = 0.34) and three individuals produced significantly narrower tools (Dolittle: beak = 14 mm, Mtool = 12.9 ± 0.07, p < 0.001; Figaro: beak = 14 mm, Mtool = 11.9 ± 0.09, p < 0.001; Pipin: beak = 13 mm, Mtool = 11.7 ± 0.09, p < 0.001; see Figure F in S1 File).

## Discussion

In this experiment, we found that Goffins switched between two different types of tool manufacturing strategies. They would either chew through the entire length of the material (full-length tool) using both mandibles like blade and anvil or adjust the length of the tool according to condition by curving it out of the material (before reaching its full-length) with a lateral slash of the upper mandible, usually while creating a curve in the cardboard (cut-out tool). We presume that the two parallel strategies most likely emerged from different, competing types of costs involved in this particular type of tool manufacture: the cognitive costs involved in attending to the distance of the reward relative to the length of the tool during manufacture as well as the physical costs involved in the manufacture of a longer tool. Just blindly chewing along the edge of the material until running out of cardboard represents low cognitive costs but high physical costs as a separate bitemark has to be placed every few millimeters in order to create the entire material stripe. Paying attention to the distance of the reward could save the physical costs of not having to make long stripes but could bear a burden in terms of attention and working memory load. The latter strategy is also likely to be more risky: if the cut-out tools turn out being too short after all the subject has to build a completely new tool.

Nevertheless, in accordance with our predictions, we found that the birds did adjust their cut-out tools depending on condition: The first self-manufactured pieces that were inserted into the apparatus on each trial were significantly longer in the far distance than in the close distance condition, suggesting that in these cases the animals did indeed attend to the lengths of their tools relative to the distance of the reward while subsequently saving unnecessary effort. Furthermore, the birds ran through the entire length of the material more often when making a tool in the far distance rather than in the close distance condition. Accounting for those tools, contrasting findings on New Caledonian crows [14, 18], and in accordance with previous research [15], the first manufactured tools that were applied to the apparatus on each trial were significantly above the minimum length required to reach the food for each the close and the far condition. In addition to this, in the trials in which the first cardboard stripe that was inserted into the box was unsuccessful, the second piece was consistently larger. This hints at the possibility that a two-stage heuristic strategy, namely adjusting an initial default behaviour as found in New Caledonian crows, may also play a limited role [18]. As the distance of the food reward had no significant effect on the length of the second tool in these cases, we do not believe that a default behaviour was corrected relative to the actual distance between the opening and the food reward but rather relative to the previous tool. The birds seem to genuinely pay attention to the food distance and were able to adjust the length of the first piece of material they inserted into the box accordingly. Nevertheless, if an object–task combination did fail, the second tool was significantly longer. This could be based on a previously developed associative learning rule such as ‘if the previous tool does not work make the next tool longer’ [18]. Resorting to default-tool based manufacture after failing with a distance based strategy may represent a risk avoidance strategy in a trial in which manufacture has already caused unrewarded effort. Note that this happened only in occasions in which the first piece of material they inserted into the box was not long enough to reach the food reward (the first tools inserted were of sufficient lengths in most trials).

The cut-out first tools inserted per trial generally tended to be larger than required in order to bridge the distance at hand. This suggests a strategy of principally building large tools like in habitually tool making birds [11, 12]. This, however, needs to be interpreted with caution as the cut-out Goffins’ tools were in fact closely around the margin of the appropriate size in the long condition (16 cm). Yet they were often close to or slightly above the length required for passing the baseline (10 cm) in the close condition (4 cm), for which five centimeter tools would have been sufficient. This indicates that, while some subjects acted differently in the two conditions (which were offered in random order) and this plasticity did save some effort, a more accurate adjustment to the actual distance to the food was only achieved in the far condition. We assume that the oversizing of tools in the close condition may have two core explanations: Firstly, the birds seemed to use two strategies in parallel, making full-length (see second green peak in Fig 3) tools alongside cut-out tools across trials. Secondly, some subjects (see description of individual performances in Results section) may have developed a ‘leftover’ tool-making habit, in this case a fixedness to a specific learnt minimum tool size for the cut-out tools (first green peak), from the baseline training before they were allowed to enter the test. Nevertheless, the birds often approached the box with a piece of material but dropped it shortly before inserting it and then went on to build a new one. In this respect, it should also be noted that the discarded tools in the close condition peaked in a ‘grey area’ (between four and five centimeters; the birds need about one centimeter to operate the tool and four centimeters to bridge the distance in order to operate the tool), only a few millimeters below functional length in three birds (Pipin, Kiwi and Fini) and that these (red) peaks were higher than the peaks of tools that were inserted by the same subjects (green peaks).

In this respect, we also address the fact that the Goffins’ inserted tools generally became longer across sessions. This trend might indicate the consolidation of an updated associative rule during the test: As making cut-out tools turned out to be risky (many discarded tools) and may have frequently caused frustration, the birds may have fallen into a strategy of generally making larger tools and thus of gradually becoming more successful. Alternatively, the birds may had become more proficient at making cardboard stripes with sufficient practice and the physical cost involved in the manufacture might have decreased continuously, prompting them to eventually adopt a similar strategy as New Caledonian crows and woodpecker finches [11, 12]. The latter species are both habitual tool users that were tested with manufacture material that was part of their habitual tool using routine. It would be interesting to test them with novel materials that require more manufacture than snipping of a branch in order to probe for a change in their original strategy.

More support for the Goffins’ ability to judge the size of their tools relative to the distance of the reward comes from the context-dependent variability with which manufactured pieces of cardboard were discarded prior to being inserted into the box. Not only were pieces of materials that were discarded by the birds significantly shorter than the distance required to reach the reward in the respective condition but shorter pieces were discarded when the reward was closer and longer pieces when the reward was further away. There is therefore the opportunity to compare the distance of the reward relative to the size of the cardboard stripe twice: once mentally before and once visually after manufacture, shortly before the stripe is inserted into the box.

In the diameter test, most of our birds failed to even insert a manufactured tool into the narrow opening. Only the female bird Fini succeeded. She only reacted differently to the width of the tools when discarding material pieces (only so in a sensible direction in the small condition). However, acknowledging that single subject designs need to be carefully planned to be telling and, given the small number of discarded tools in the large condition (N = 5) we refrain from making any conclusions based on these data.

It is safe to argue that the width of the cardboard stripe was constrained by the beak morphology of the respective subject: The lower edge of the upper mandible of the Goffin’s cockatoo takes an about 90° steep curved angle from the beak tip to the corner of the mouth. The edge of the cardboard block is pressed into the deepest possible point of that curve during manufacture for support. Meanwhile the beak tip was used to cut through the material while pushing against it from the other side with the lower mandible. Using this manufacturing technique, the width of the cardboard tools is largely restricted by the distance from the respective bird’s beak tip to the deepest part of the curve on the lower edge of the upper mandible. Like many parrots, Goffins have a slight dimorphism in body weight and particularly in beak size with males being slightly heavier and having larger beaks [34]. When we measured the aforementioned distance in our subjects we indeed found that this part of the beak was several millimetres larger in all males we tested than in the female Fini. We found that two males generally built marginally larger tools (by less than a millimetre) than their beak size, Fini build them on average just as wide as her beak size and three more males built them slightly (up to 2mm) smaller on average than their beak but still too wide to fit through the narrow opening in the diameter condition. As the curve in their upper mandible restricts the maximum width of the tool, it is not possible for the birds to build the tool wider than this beak measurement, at least not as long as this particular building technique is employed. Nevertheless, while they should ergonomically be able to build them thinner, this is most likely not very convenient for them as the deep point of the beak’s curve can then not be used as support to keep the material in place while cutting it at the front of the beak. They also failed to innovate alternative techniques to support the material (other than in the curve of their upper mandible) that would have allowed for making slimmer tools. This could represent a cognitive barrier within the Goffin’s tool making abilities.

In conclusion, our findings support previous studies on Goffins showing that they can not only select [26, 27] but create different tools depending on the task at hand [28].

They show very high success rates using self-manufactured pieces of material and could adjust their cut-out tools to different conditions. They seem to be able to judge when a cut-out tool is too short for success by comparing the length of their tool relative to the condition not necessarily during the manufacture process but shortly before bringing it in contact with the box. Nevertheless, their sensitivity to exact goal distances seems to be limited: while they could accurately adjust the lengths of their tools to the reward distance in the far condition, they failed to do so in the close condition. Whether this is an effect of associative learning or a fixedness from our training remains subject to future research.

Furthermore, they do not adjust the width of a tool to function. While this could be a cognitive limit within their tool innovation abilities, the Goffin’s beak morphology and its manufacturing technique for cardboard tools suggests an ergonomical reason. Upcoming studies featuring different types of materials that require less restrictive handling will be able to follow this up.

## Supporting information

S1 File Supplementary information: Contains details of both the training procedure as well as the results in Microsoft word. (DOCX)

S2 File Raw data: Contains the data obtained for the experiments in Microsoft excel. (XLSX)
