# Supplementary material for: Tool making cockatoos adjust the lengths but not the widths of their tools to function
Source: PLoS One. 2018 Nov 7;13(11):e0205429. doi: 10.1371/journal.pone.0205429 (PMC6221259; doi:10.1371/journal.pone.0205429)
Supplement: S1 File — (DOCX) [file pone.0205429.s001.docx]

**S1 Supplementary information**

**Tool making cockatoos adjust the length but not the width of their tools to function**

A.M.I. Auersperg^1*†^, C. Köck^2†^, M. O’Hara^1^, L. Huber^1^

^1^Messerli Research Institute, University of Veterinary Medicine Vienna, Medical University of Vienna, University of Vienna, Veterinärplatz 1, 1210 Vienna, Austria

^2^Department of Cognitive Biology University of Vienna, Althanstr. 14, 1090 Vienna, Austria

^*^Corresponding Author: [alice.auersperg@vetmeduni.ac.at](mailto:alice.auersperg@vetmeduni.ac.at)

Phone: +43 676 9390392

^†^ shared first authorship

**Pre-training**

Before testing started only two subjects had previously made cardboard tools, we therefore trained four more birds to increase our sample.

*Training of naïve subjects*

After being allowed to retrieve food from the apparatus using a ready-made tool, the four subjects previously naïve in cardboard tool manufacture (Fini, Pipin, Kiwi & Konrad) were offered a cardboard block (15 x 6 cm) in front of the baited apparatus from the previous study [14]. If subjects successfully made cardboard tools they received up to nine additional trials within the same session (with the same procedure). If they failed to do so within 10 min of the same trial they received another session on the next testing day. If a bird failed to manufacture a cardboard tool within five sessions, a demonstrator bird (Figaro or Dolittle) made and used three tools in succession while the observer waited in an adjacent parrot cage. Each naïve bird was thereafter given the opportunity again to make cardboard tools themselves (once again for 10 minutes) for a maximum of five demo-followed sessions. Subjects that still failed to make cardboard tools were presented with cardboard sheets that were perforated along the edge of the material (with perforations alongside the longer edge of the cardboard, in a 1 cm interval) prior to being retested with the original material (until they became continuously successful) .

Fini was successful for a single time in session five (before demos or pre-punched sheets). She thereafter received demonstrations. After succeeding to make a functional tool each for a single time after demo sessions one and two she became continuously successful (10 consecutive trials) after demo session three.

Kiwi Pipin and Konrad did not succeed before receiving pre-punched cardboard sheets. All made functional tools from regular cardboard sheets from session one after experiencing pre-punched sheets, Kiwi became continuously successful at doing so from session three (S1: 1 tool; S2: 1 tools; S3: 10 tools), Pipin from session 6 ( S1: 2 tools; S2: 1 tool; S3: 1 tool; S4: 3 tools; S5: 3 tools; S6:10 tools) and Konrad from session 7 ( S1: 1 tool; S2: 1 tool; S3: 1 tool; S4: 1 tool; S5: 1 tool, S6: 4 tools; S7: 10 tools).

*Reminder trials for cardboard-tool-making subjects*

Subjects that already had experience with cardboard tool manufacture were given 2 sessions of reminder trials (1 session consisted of 10 trials) to test whether they still made tools out of cardboard after a testing pause of several months [14]. All subjects were continuously successful.

**Supplementary Results**

**Results Test**

Due to methodological issues measurements of discarded and unsuccessful tools are lacking for some trials. For Fini measurements in 14.6% (17 trials) are missing, for Figaro 13.33% (16 trials), for Kiwi 2.5% (3 trials), for Konrad and Pipin each 1.67% (2 trials) are unaccounted for. In total these make up 5.56 % of all trials in which full data was not available. We omitted these trials from the analysis.


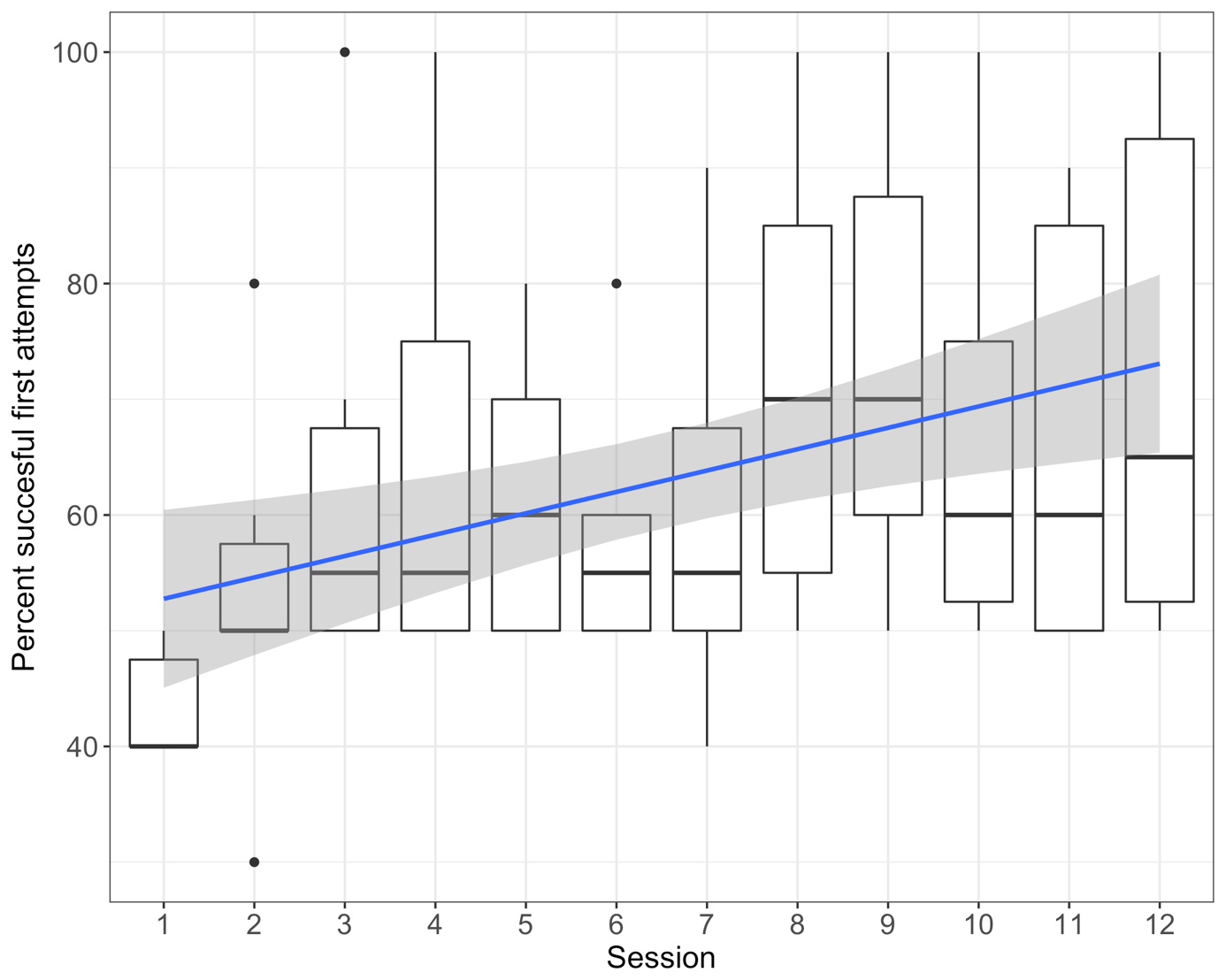


**Figure A**: Percent of successful first attempts over sessions; blue line represents the fitted regression line; shaded area represents 95% confidence region; bold horizontal lines indicate median values, boxes span the first to third quartiles and whiskers represent 95% confidence intervals

**Table A**: Multiple comparisons of overall success rates in different conditions. P values are adjusted for multiple comparisons.

Model: *first correct choices ~ Condition + Session + (1|Subject)*

Significance codes: ‘***’ for *p* < 0.001; ‘**’ for *p* < 0.01; ‘*’ for *p* < 0.05; '.' for *p* < 0.1

|  | **β** | **Std. Error** | **z** | **p** | **Sig.** |
| --- | --- | --- | --- | --- | --- |
| Large vs. Far | 28.91 | 0.476 | 6.071 | <0.001 | *** |
| Near vs. Far | 0.783 | 0.261 | 2.997 | 0.013 | * |
| Small vs. Far | -37.33 | 0.375 | -9.945 | <0.001 | *** |
| Near vs. Large | -21.08 | 0.485 | -4.344 | <0.001 | *** |
| Small vs. Large | -66.25 | 0.588 | -11.261 | <0.001 | *** |
| Small vs. Near | -45.17 | 0.396 | -11.412 | <0.001 | *** |

**Table B**: detailed contrast results for levels of tool application and distance to reward. P values are adjusted for multiple comparisons.

Model: $\sqrt{Length}$ *~ Distance/Use + Session + (1|Subject)*

Significance codes: ‘***’ for *p* < 0.001; ‘**’ for *p* < 0.01; ‘*’ for *p* < 0.05; '.' for *p* < 0.1

|  | **β** | **Std. Error** | **z** | **p** | **Sig.** |
| --- | --- | --- | --- | --- | --- |
| 40mm Discarded vs. 160mm Discarded | -10.885 | 0.3750 | -2.903 | 0.0191 | * |
| 160mm Used vs. 160mm.Discarded | 22.852 | 0.3171 | 7.207 | <0.001 | *** |
| 40mm Used vs. 160mm Discarded | 15.618 | 0.3225 | 4.843 | <0.001 | *** |
| 160mm Used vs. 40mm Discarded | 33.737 | 0.3391 | 9.949 | <0.001 | *** |
| 40mm Used vs. 40mm Discarded | 26.503 | 0.3324 | 7.973 | <0.001 | *** |
| 40mm Used vs. 160mm Used | -0.7234 | 0.2588 | -2.796 | 0.0261 | * |


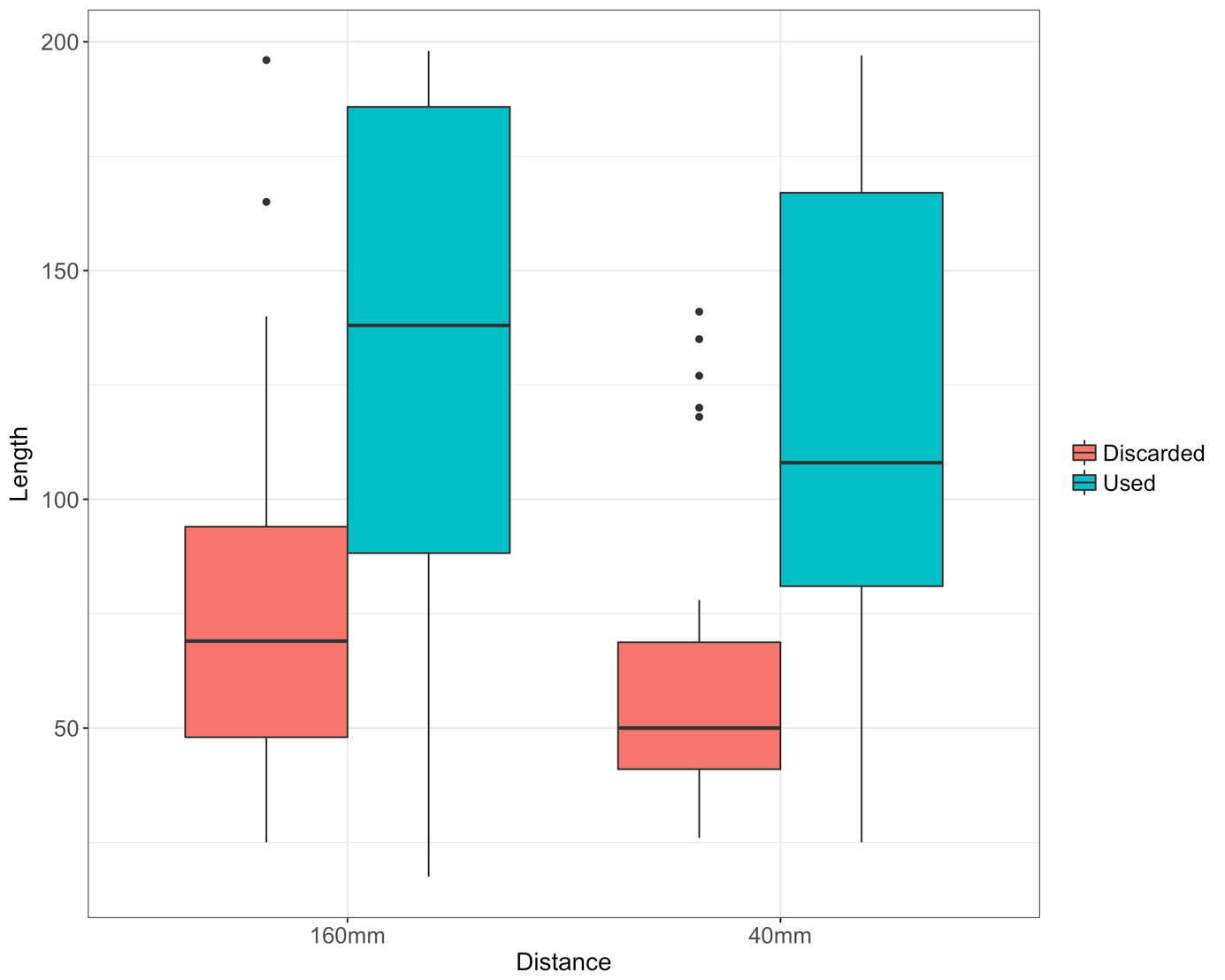


**Figure B**: Length of employed and discarded tools in different distance conditions; bold horizontal lines indicate median values, boxes span the first to third quartiles and whiskers represent 95% confidence intervals


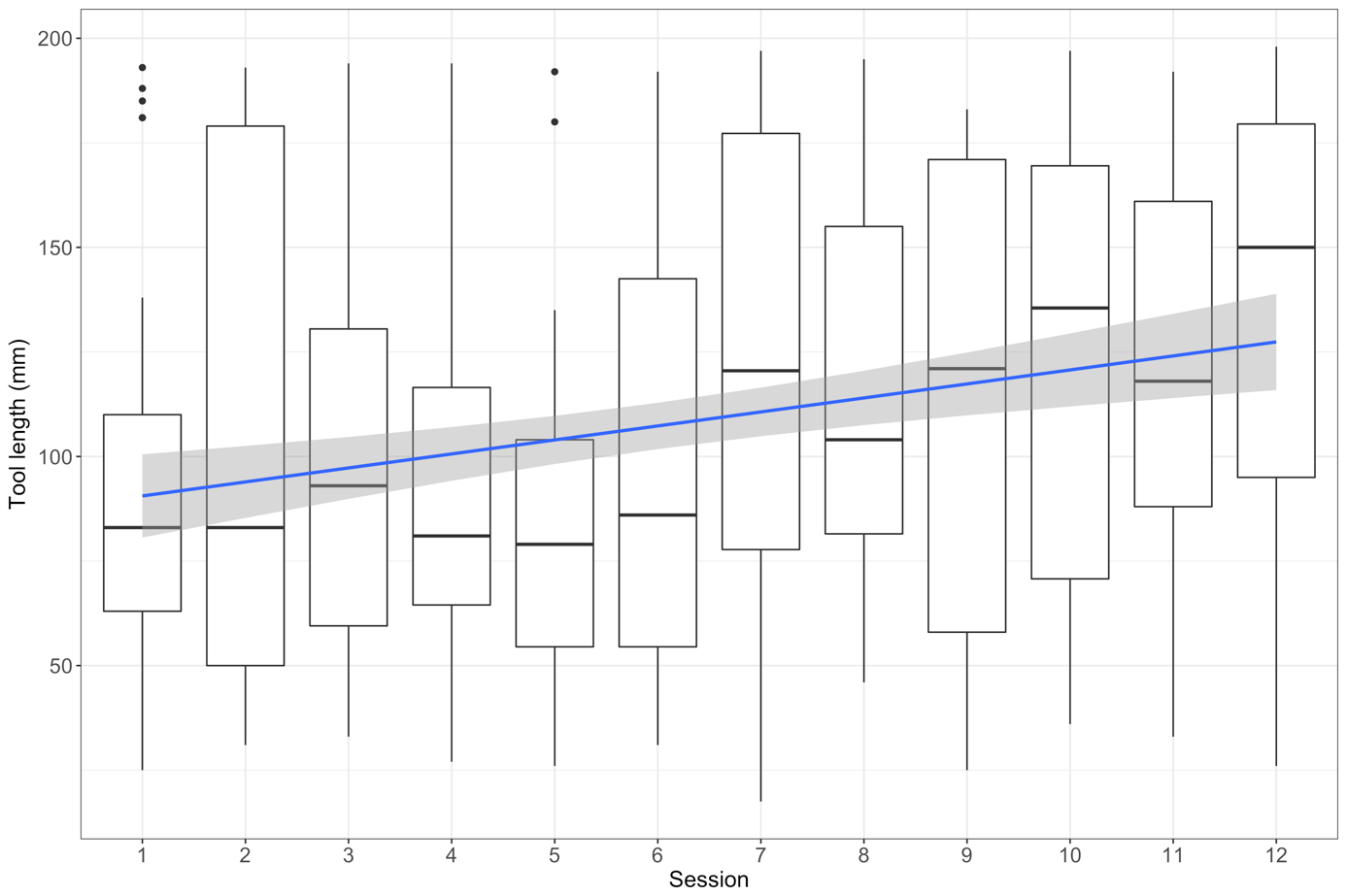


**Figure C**: Length of tools (discarded and used, smaller than 200mm) in the distance test over sessions; blue line represents the fitted regression line; shaded area represent 95% confidence region; bold horizontal lines indicate median values, boxes span the first to third quartiles and whiskers represent 95% confidence intervals


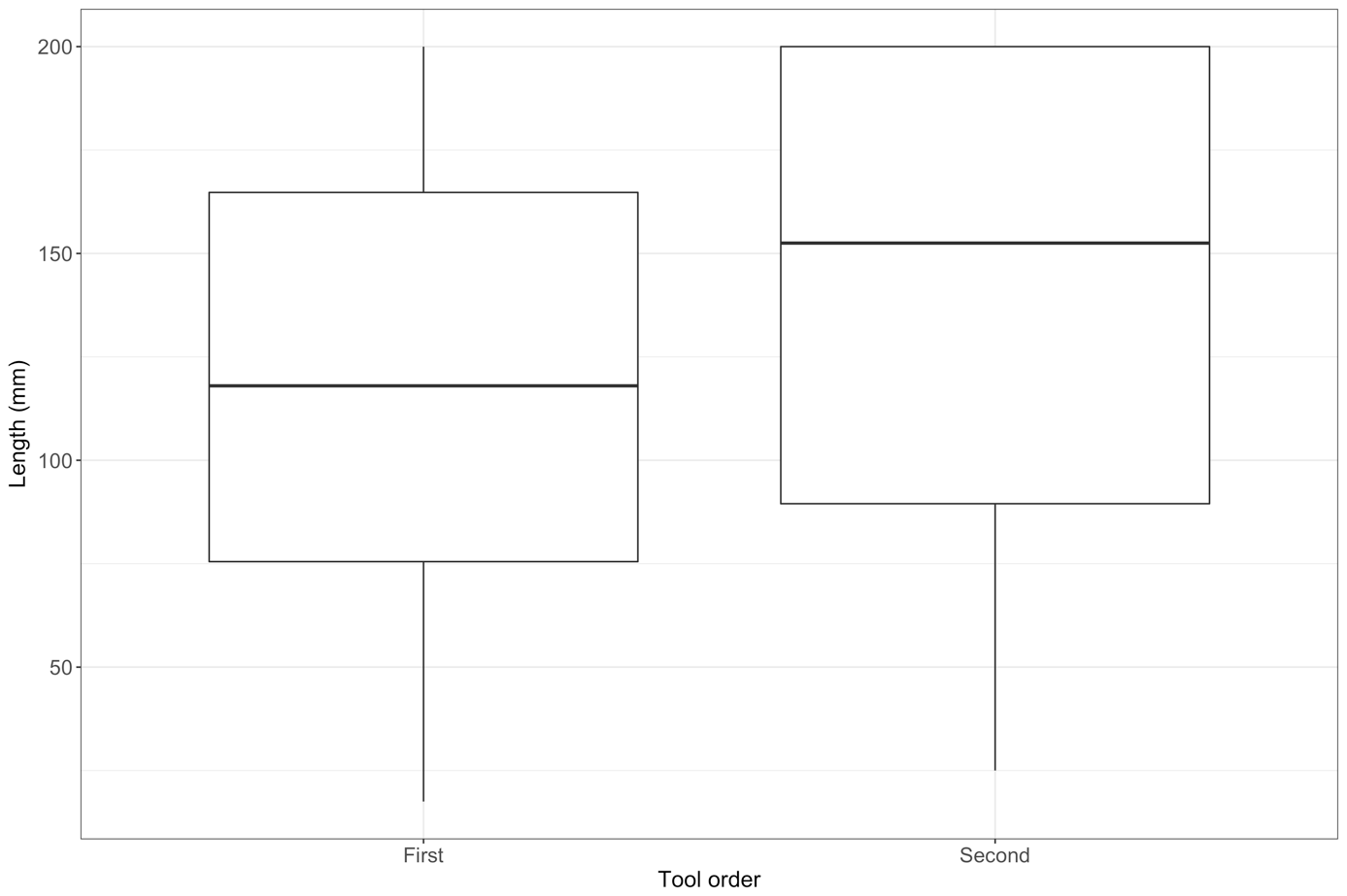


**Figure D**: differences in tool length (in mm) between unsuccessful first tools and second employed tools; bold horizontal lines indicate median values, boxes span the first to third quartiles and whiskers represent 95% confidence intervals

748


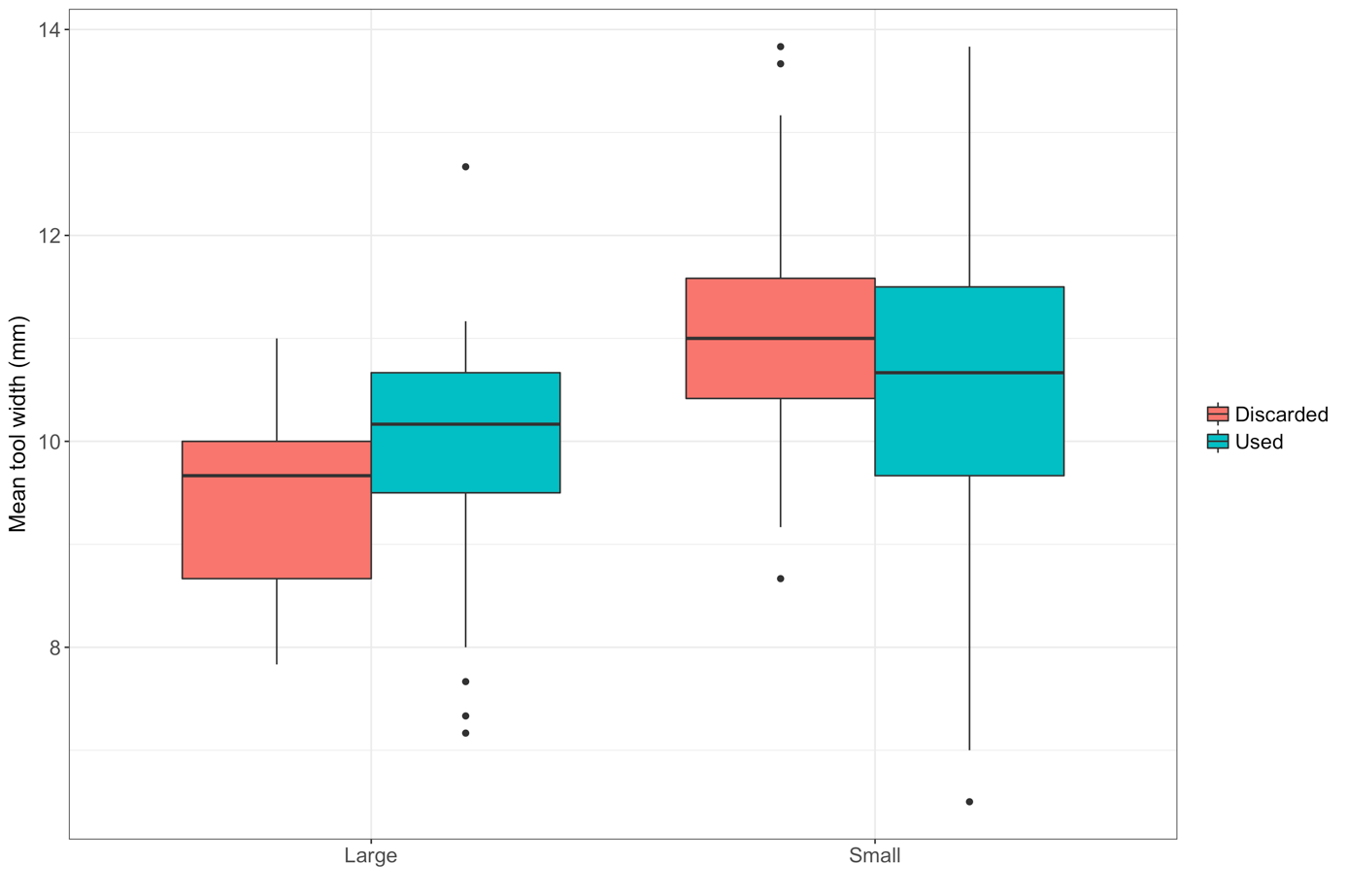


**Figure E**: Mean width of discarded and used tools of Fini with large holes and with small holes; bold horizontal lines indicate median values, boxes span the first to third quartiles and whiskers represent 95% confidence intervals


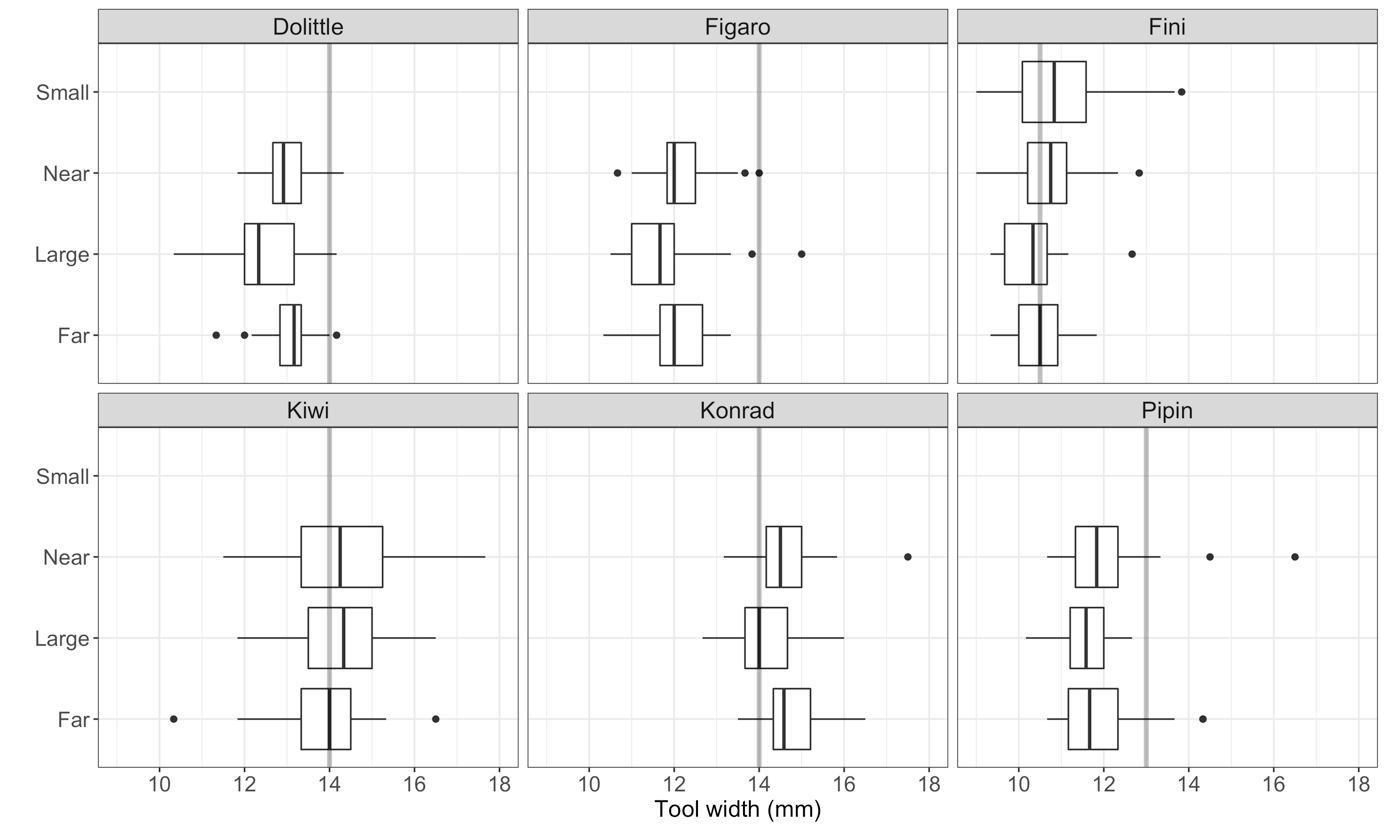


**Figure F**: Width of employed tools (in mm) in different conditions for each subject compared to the distance between the tip of the beak and the horizontal part of the upper mandible (gray vertical line); bold horizontal lines indicate median values, boxes span the first to third quartiles and whiskers represent 95% confidence intervals
